# How i do it: a survey of practice patterns in robot-assisted radical prostatectomy

**DOI:** 10.1007/s11701-026-04080-3

**Published:** 2026-10-27

**Authors:** Narmina Khanmammadova, Johnny Wang, Tuan Thanh Nguyen, Dat Tien Nguyen, Mohammad Abubakr, Jade Vanta, Kristene Myklak, Daniel Jiang, Sohrab N. Ali, Jacob Basilius, Mohammed Shahait, David I. Lee

**Affiliations:** 1 Department of Urology, University of California, 3800 W Chapman Ave, Suite 7200, Orange, Irvine, CA 92868 USA; 2 School of Medicine, University of California, Riverside, CA USA; 3 Department of Urology, University of Medicine and Pharmacy at Ho Chi Minh City, Ho Chi Minh, Vietnam; 4 Department of Urological Oncology Surgery, Binh Dan Hospital, Ho Chi Minh, Vietnam; 5 Department of Urology, Division of Urologic Oncology, City of Hope Comprehensive Cancer Center, CA, USA

**Keywords:** Robot-assisted radical prostatectomy, Prostate cancer, Practice patterns

## Abstract

Despite the global availability and utilization of robot-assisted radical prostatectomy (RARP), it remains a technically complex operation, with wide variation in training paradigms, surgical technique, and perioperative management. This study examines international RARP practices and aims to identify trends and gaps in clinical practice. A multiple-choice questionnaire consisting of 32 questions was distributed between January and July 2023. A total of 102 responses were received. Responses with ≥ 5 questions with missing data (*n* = 8) were excluded. Among 94 participants, 91.5% were male, 69.1% practiced outside the United States (US), and 72.3% were fellowship-trained. Most surgeons (79.8%) reported performing ≥ 3 RARPs monthly. About 75% of surgeons reported performing ≥ 90% of their radical prostatectomies (RP) robotically. Seven (7.45%) respondents who reported performing RPs only via laparoscopic or open approach were excluded from subsequent analyses. Regarding the surgical technique, transperitoneal anterior approach was the most preferred (62.1%). For nerve-sparing, the combined antegrade-retrograde approach (43.7%) and the antegrade-only approaches (36.8%) were most common. Approximately half of respondents (51.7%) preferred cut-and-suture ligation for dorsal venous complex control. For pelvic lymph node dissection, standard (44.8%) or limited (29.9%) templates were preferred over the extended (24.1%) template. The most common method for vascular control was clips for vascular pedicle ligation (79.3%). Of the continence-preserving techniques, most respondents agreed on the importance of maximal urethral length preservation (93.1%), bladder neck preservation (80.5%), and posterior reconstruction (64.4%). Postoperatively, most surgeons endorsed early mobilization (92%), early oral nutrition (85.1%), and non-opioid oral analgesia (57.5%). All US-based surgeons reported discharging ≥ 70% of patients on postoperative day 1, whereas discharge patterns varied among non-US surgeons. There are significant global variations in the practice of RARP. These findings point to the potential utility of consensus-driven, evidence-based guidelines to optimize RARP practices worldwide.

## Introduction

In the last two decades, robot-assisted radical prostatectomy (RARP) has become the most common approach for radical prostatectomy (RP) [1–3]. Its demonstrated advantages include improved visualization, ergonomics, and perioperative outcomes compared to open (ORP) and laparoscopic radical prostatectomy (LRP) [4–7]. Yet, RARP remains a technically complex operation that can be hugely impactful to a patient’s functional outcomes and quality of life.

In tandem with the increased number of experienced surgeons performing the surgery and an improved understanding of the anatomy of the prostate and associated structures, many technical modifications have been proposed to each step of the procedure in hopes of enhancing outcomes [8–14].

Adoption of these modifications varies among surgeons and is influenced by factors including each surgeon’s training and experience, availability and cost of equipment, and patient and tumor characteristics. However, little is known about the diversity of RARP practices globally. Characterizing these patterns may assist in determining which techniques and practices shape how RARP is performed in the real world.

We conducted a cross-sectional study to capture the training status of surgeons and their preferred practices for RARP. By identifying common trends and gaps in practice, this study seeks to clarify the ever-evolving landscape of RARP and highlights potential targets for further study and refinement.

## Methods

A survey instrument consisting of 32 questions in the English language was developed by a group of urologists with extensive experience in minimally invasive urologic surgery and RARP. The survey was designed to characterize self-reported experiences and preferences rather than to assess technical performance or clinical outcomes. Questions were initially drafted by members of the research team (N.K., T.T.N., D.T.N.), reviewed by the senior authors (S.N.A., M.S., D.I.L.), and refined through group discussion until consensus was achieved. Although the final instrument did not undergo formal validation, all included questions were designed to be concise and directly applicable to RARP workflows.

The survey consisted of questions addressing domains of basic demographics (i.e., age, gender, race, and location), training (fellowship type, RARP-specific training opportunities, RARP techniques learned in training), clinical practice (i.e., subspecialization, years in practice, practice setting, RARP case volume), surgical technique (anatomical approach, nerve-sparing, techniques for continence, dorsal venous complex [DVC] control, pelvic lymph node dissection [PLND]), surgical setup and instrumentation (fourth robotic arm, bedside assistant, insufflation, hemostatic agents), and perioperative care (enhanced recovery after surgery [ERAS] protocol adherence, discharge timing, catheter management, drain placement, cystogram use).

Following approval from the Institutional Review Board (protocol no. 2402), the survey was distributed between January 2023 and July 2023 to practicing urologists through social media and in-person during international urology conferences. Additionally, we distributed the survey via email to members of the Endourological Society, including two rounds of reminder notifications. The REDCap (Vanderbilt University, Nashville, TN) web platform was used to collect and store data securely. We received responses from 102 participants. Those without data for ≥ 5 questions (*n* = 8) were excluded, with the final cohort consisting of data from 94 participants. Data were analyzed using IBM SPSS Statistics for Windows, Version 29.0 (IBM Corp., Armonk, NY, USA). Results are presented as numbers and percentages. Questions with multiple choice answer formats have percentages that sum to more than 100%.

## Results

### Background, training, and practice

**Fig. 1:**
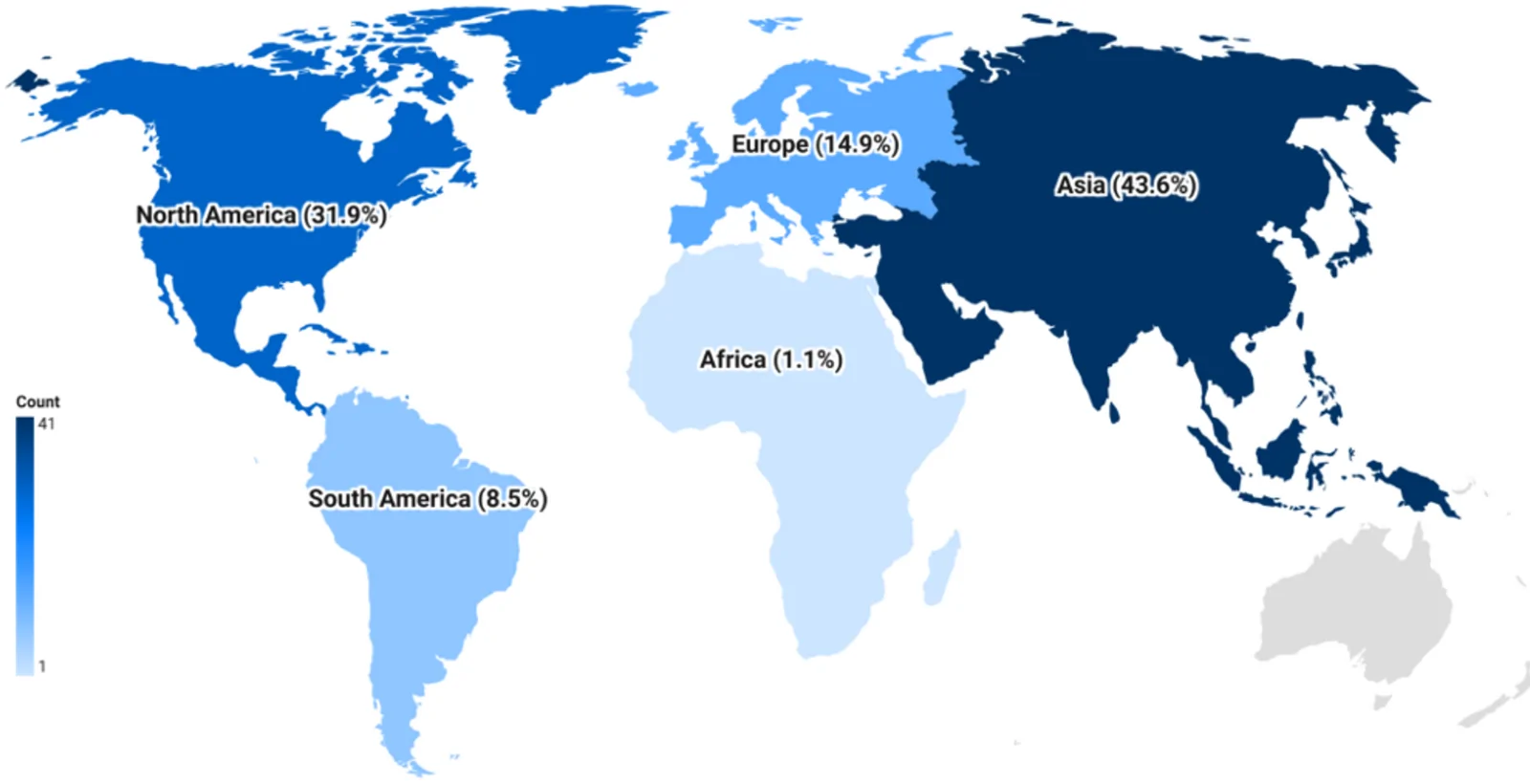
Geographic distribution of participating urologists

Demographics and practice characteristics are shown in Table 1. Of the 94 respondents, most were male (91.5%) and practiced outside the United States (69.1%). A map indicating country of practice for all respondents is shown in Fig. 1. Experience level varied: 17% had 0–5 years in practice, 17% had 6–10 years, 24.5% had 11–15 years, 19.1% had 16–20 years, and 22.3% had > 20 years. The most common practice setting was academic or university hospital (72.3%). Respondents underwent fellowship training in robotic and laparoscopic surgery (41.2%), urologic oncology (36.8%), or endourology (16.2%). Respondents identified their specialty focus most commonly as urologic oncology (69.1%), followed by endourology (16%) and general urology (13.8%).

**Table 1:** Demographics, training, and practice characteristics

Variable	Responses, *n* (%)
Gender	
Male	90 (91.5%)
Female	8 (8.5%)
Years in clinical practice	
0–5 years	16 (17.0%)
6–10 years	16 (17.0%)
11–15 years	23 (24.5%)
16–20 years	18 (19.1%)
>20 years	21 (22.3%)
Fellowship training	
Yes	68 (72.3%)
No	26 (27.7%)
Fellowship type	
Robotic & Laparoscopic Surgery	28 (41.2%)
Urologic Oncology	25 (36.8%)
Endourology	11 (16.2%)
Other	4 (5.9%)
Practice type	
Urologic oncology	65 (69.1%)
Endourology	15 (16.0%)
General urology	13 (13.8%)
Other	1 (1.1%)
Practice setting	
Academic hospital	68 (72.3%)
Private practice	16 (17.0%)
Non-academic hospital	9 (9.6%)
Other	1 (1.1%)
Access to daVinci Robot	
Yes	87 (92.6%)
No	7 (7.4%)
RARP training	
During residency	32 (34.0%)
During fellowship	46 (48.9%)
Short-term training (<3 months)	22 (23.4%)
In-person instructional courses	25 (26.6%)
Online instructional courses	18 (19.1%)
Independent reading	12 (12.8%)
Surgical videos	32 (34.0%)
No specified training	9 (9.6%)
Radical prostatectomies performed per month	
None	1 (1.1%)
1-2	18 (19.1%)
3-5	44 (46.8%)
6-10	14 (14.9%)
>10	17 (18.1%)

JP = Jackson-Pratt, ERAS = Enhanced Recovery After Surgery, PO = by mouth, IV = intravenous, IQ pump = intravenous pain management pump

Nearly all (92.6%) reported access to a daVinci^®^ robotic platform. Formal training in RARP occurred mainly during fellowship (48.9%) and/or residency (34%). Additional training experiences included short-term programs (< 3 months) (23.4%), in-person instructional courses (26.6%), and surgical videos (34%). In terms of case volume, 79.8% reported performing ≥ 3 RARPs per month. RARP was overwhelmingly preferred over ORP and LRP, as it was used for 100% of radical prostatectomies by 62.4% and for ≥ 90% by 75.3%. ORP and LRP were never used by 66 (71.0%) and 73 (78.5%) respondents, respectively. Seven respondents reported performing 0% of their RPs robotically and were excluded from subsequent analyses.

### Techniques in RARP

**Fig. 2:**
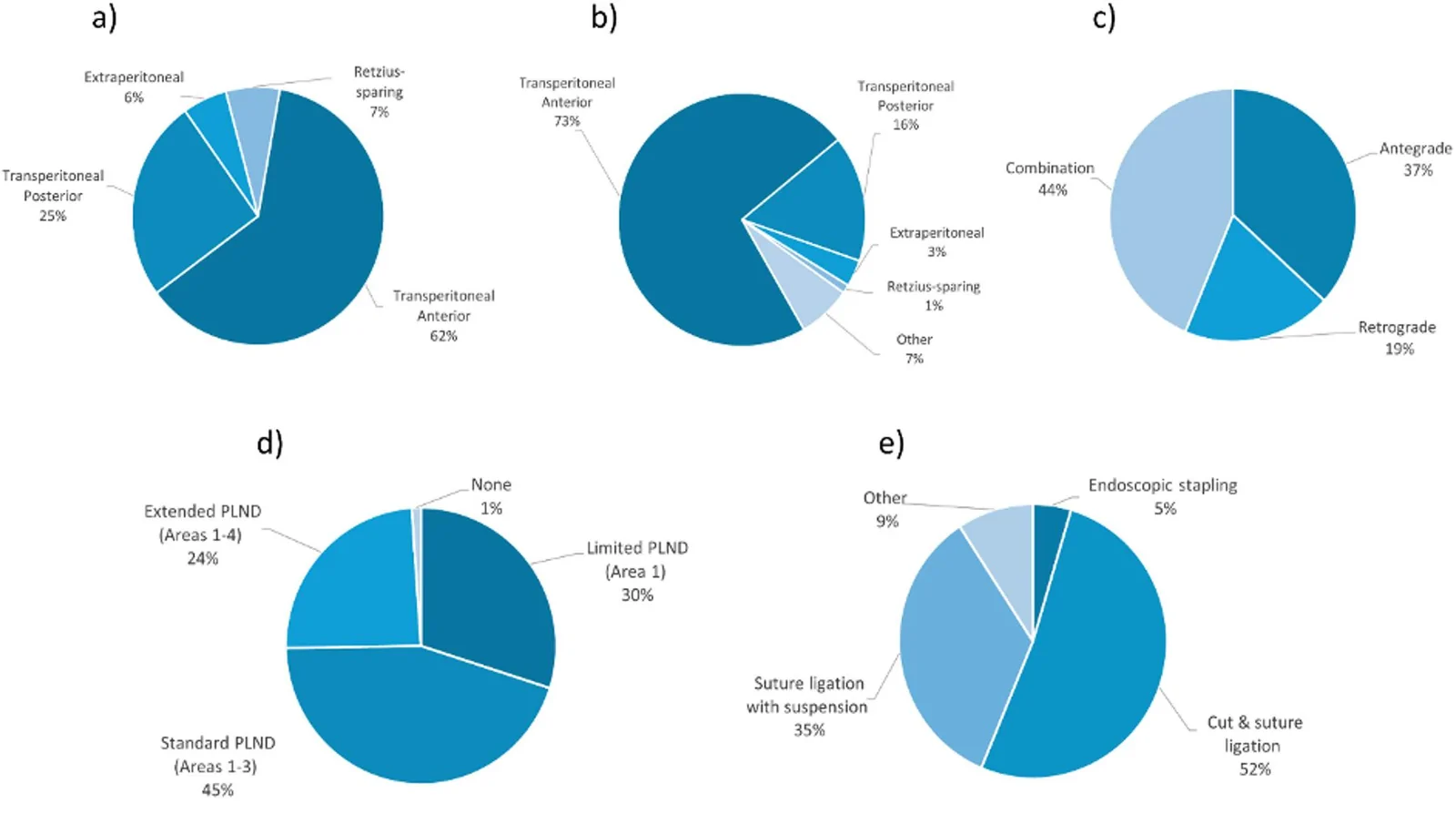
Technical preferences during RARP: (**a**) access used in practice; (**b**) access learned in training; (**c**) approaches to nerve-sparing dissection; (**d**) PLND template; (**e**) method of DVC control

Technical preferences of participating surgeons are characterized in Fig. 2. The transperitoneal anterior approach was the most preferred (62.1%) and most frequently learned (72.4%) access for RARP. Overall, among 87 available responses, 58 (66.7%) reported that their preferred approach during practice remained the same as the approach to which they were exposed during training. Forty-five (77.6%) learned the transperitoneal anterior approach and reported this as their preferred approach during practice. Twelve (20.7%) learned the transperitoneal posterior approach and reported this as their preferred approach during practice. One (1.7%) reported learning the extraperitoneal approach during training and also reported this as a preferred approach during practice.

The most common approaches to nerve-sparing were the combined antegrade-retrograde approach (43.7%) or the antegrade-only approach (36.8%). The preferred method of dorsal venous complex control was cut-and-suture ligation (51.7%) or suspension suture (34.5%). For pelvic lymph node dissection (PLND), respondents preferred standard (44.8%) or limited (29.9%) dissection templates over the extended (24.1%) template. There were also varied strategies to achieve nerve sparing and improved continence. During nerve sparing, the most common method for vascular control was clips for vascular pedicle ligation (79.3%), followed by electrocautery (36.8%) and suture ligatures (20.7%). Of the continence-preserving techniques, most respondents agreed on maximal urethral length preservation (93.1%), bladder neck preservation (80.5%), and posterior reconstruction (64.4%). Less common technical points included anterior suspension sutures (29.9%), high nerve release (37.9%), Hood technique (25.3%), and Retzius-sparing approach (12.6%).

### Surgical setup and instrumentation

A fourth robotic arm was used by 94.3% of respondents. Bedside assistants were most often advanced practice providers (APPs) (43.7%) or fellows/residents (41.4%) and were preferentially positioned on the patient’s right side (59.8%). Respondents used standard (49.4%) or AirSeal^®^ (49.4%) insufflation systems. Standard pressure pneumoperitoneum (12–15 mmHg) (70.1%) was most common, with a smaller proportion (24.1%) using low pressure. Barbed suture was the predominant suture of choice for vesicourethral anastomosis (88.3%). Almost half of respondents reported regular use of hemostatic agents, including Surgicel^®^ (20.7%), Floseal^®^ (16.1%), and Tisseel^®^ (8.0%). These results are summarized in Table 2.

**Table 2:** Surgical setup and instrumentation

Variable	Responses, *n* (%)
Insufflation system used	
Standard	43 (49.4%)
AirSeal	43 (49.4%)
Other	1 (1.1%)
Preferred insufflation pressure	
Standard (12–15 mmHg)	61 (70.1%)
Low (10–11 mmHg)	21 (24.1%)
Ultra-low (< 10 mmHg)	5 (5.7%)
Bedside assistant type	
APP	38 (43.7%)
Fellow/resident	36 (41.4%)
Partner/urologist	13 (14.9%)
Assistant position	
Right side	52 (59.8%)
Left side	35 (40.2%)
Use of fourth arm	
Yes	82 (94.3%)
No	5 (5.7%)
Suture type used for anastomosis	
Barbed	80 (92.0%)
Monofilament	5 (5.7%)
Braided	2 (2.3%)
Use of hemostatic agents	
Yes	42 (48.3%)
Surgicel	18 (20.7%)
Floseal	14 (16.1%)
Tisseel	7 (8.0%)
Other	11 (12.6%)
No	43 (49.4%)

APP = advanced practice provider

### Perioperative management

As seen in Table 3, the implementation of enhanced recovery after surgery (ERAS) principles varied among respondents. Preoperative administration of oral hydration (25.3%), acetaminophen (20.7%), and gabapentin (5.7%) were uncommon. Most did not use bowel preparation preoperatively (69%). During surgery, local and/or regional anesthesia was utilized by 64.4%. Postoperatively, early mobilization (92%), early oral nutrition (85.1%), and non-opioid oral analgesia (57.5%) were agreed upon by most. Postoperative cystography was performed routinely by some respondents (29.9%).

**Table 3:** Details of perioperative care

Variable	Responses, *n* (%)
Postoperative cystogram performed	
Yes	26 (29.9%)
No	61 (70.1%)
Use of surgical drain	
Yes	51 (58.6%)
No	36 (41.4%)
Utilization of ERAS protocol	
No bowel preparation	60 (69.0%)
Local anesthesia	42 (48.3%)
Regional anesthesia	14 (16.1%)
Non-opioid oral analgesia after surgery	50 (57.5%)
Early oral nutrition after surgery	74 (85.1%)
Early mobilization after surgery	80 (92.0%)
Preoperative PO acetaminophen	18 (20.7%)
Preoperative PO anticholinergic	4 (4.6%)
Preoperative PO gabapentin	5 (5.7%)
Preoperative PO fluids	22 (25.3%)
Postoperative IV acetaminophen	25 (28.7%)
Postoperative IQ pump	3 (3.4%)
Other	6 (6.9%)
Timing of postoperative catheter removal	
< 5 days	6 (6.4%)
5–7 days	58 (61.7%)
> 7 days	30 (31.9%)

JP = Jackson-Pratt, ERAS = Enhanced Recovery After Surgery, PO = by mouth, IV = intravenous, IQ pump = intravenous pain management pump

**Fig. 3:**
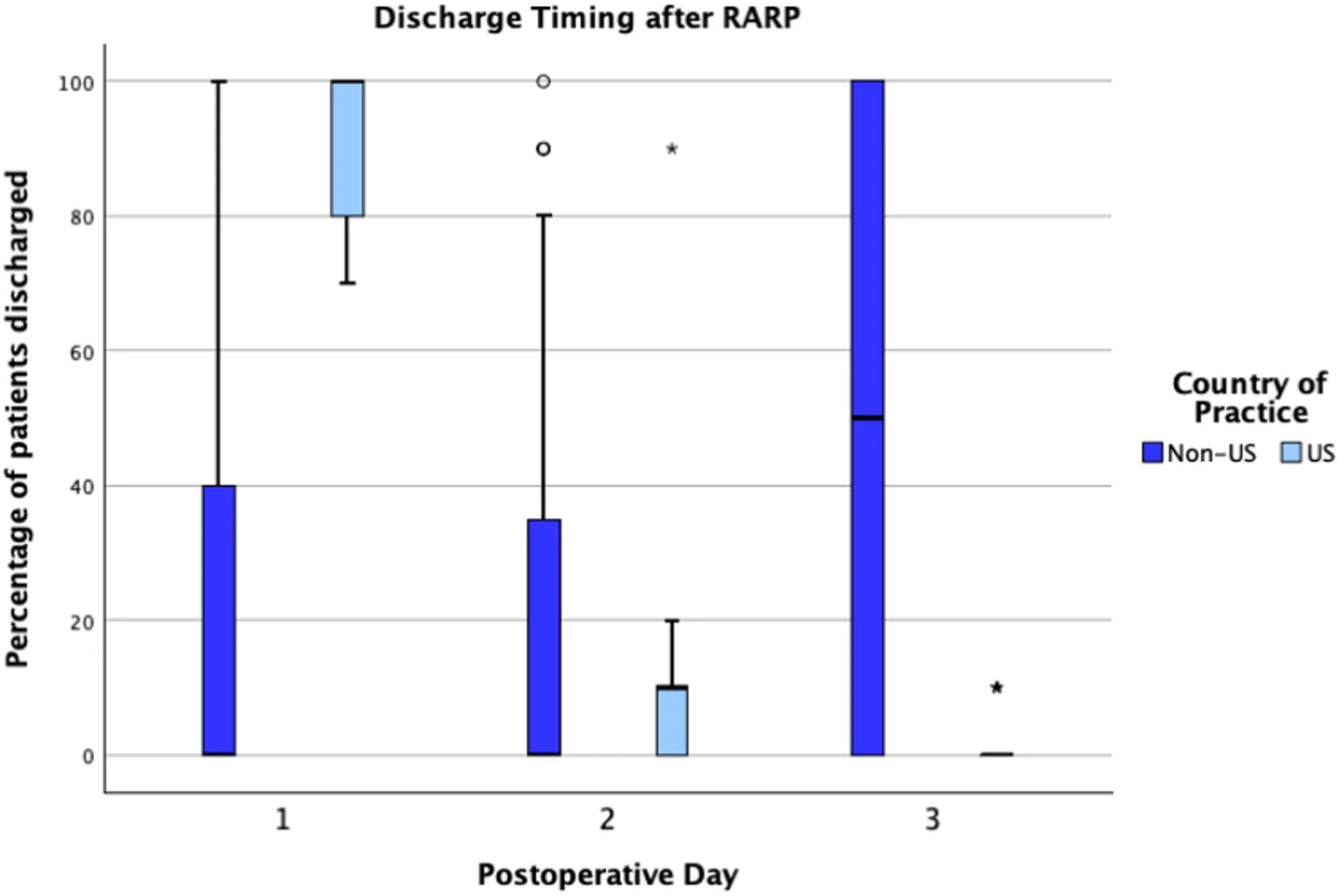
Surgeon-reported discharge rates after RARP

Discharge patterns after RARP stratified by US versus non-US are shown in Fig. 3. Notably, all surgeons based in the US discharged 70% or more of their patients on postoperative day (POD) 1, with 20.7% of surgeons reporting that all patients are discharged by POD 1. Conversely, 27.6% of non-US surgeons reported that all of their patients stay until day 3 or later. Catheter removal was typically performed between postoperative days 5–7 (63.2%).

## Discussion

RARP is one of the most common major urologic surgeries and continues to evolve in practice. Sharing surgeon experiences can identify opportunities for further optimization. This study characterizes the practices of 94 urologists worldwide. We report heterogeneity in surgical techniques and perioperative management, including interesting differences in approaches to access, nerve-sparing, PLND, techniques for continence, and discharge practices.

Upon its introduction in the early 2000s, RARP was rapidly adopted by urologists in the US [15]. By 2011–2012, it had outgrown both ORP and LRP, comprising over 70% of RPs performed in the US [16]. In contrast, uptake outside the US lagged due to high costs and limited access [17]. Yet, urologists in the Middle East and Asia, despite less exposure, were more likely than their Western peers to endorse robotics as the gold standard for prostatectomy, cystectomy, and nephrectomy [18]. In this study, 92.6% of respondents, with representation across South America, the Middle East, Africa, and Asia, reported access to a daVinci^®^ robot. Moreover, 62.4% reported exclusively performing RARPs over ORP and LRP.

Though widely disseminated, robotic skills training remains inconsistent. Half of our respondents learned RARP in fellowship, and only a third received training in residency. Recent data have shown that most residents perform less than 10 full RARPs as the primary console surgeon during training [19]. Furthermore, many respondents sought training through mini-fellowships (duration < 3 months), instructional courses, and video review, reflecting the self-directed model of robotic surgery education. Given the growing technical complexity of RARP, more standardized curricula may be warranted.

The transperitoneal anterior access was described in the very first reports of RARP and is now considered the traditional access [20]. It remains by far the preferred and most frequently learned approach for urologists in our study. Of the novel access options, the Retzius-sparing approach was notably uncommon, as it was preferred by 6.9% and learned by only 6.4% during training. Interestingly, 66.7% of the surgeons reported preferring the same approach they learned during training. This finding further underscores the importance of continued education and technical adaptability as RARP techniques continue to evolve, with potential implications for patient outcomes.

Nerve-sparing remains a technically challenging step of great interest to patients and surgeons alike. Antegrade or combined antegrade-retrograde approaches were favored over retrograde dissection. In the modern era, antegrade dissection is most common, as it offers more working space and better visualization of dissection planes [21]. On the other hand, retrograde dissection permits early release of the neurovascular bundles and may reduce traction injury, but evidence is mixed regarding functional outcomes [21].

When assessing principles for improved continence, respondents emphasized maximal urethral length preservation (93.1%) and preservation of the bladder neck (80.5%), both associated with improved early and late continence [22, 23]. Posterior reconstruction (also known as the Rocco stitch) was common in our cohort consisting mostly of surgeons with > 10 years in practice, suggesting that its perceived benefit in facilitating the vesicourethral anastomosis remains relevant even for experienced operators. Additionally, the Hood technique, which spares the anterior periurethral structures for faster recovery of continence, was used by 25.3% of respondents [24]. The limited preference for this approach could be attributed to its difficulty and the reluctance to use it in patients with high-risk disease and anterior or apical tumors.

PLND practices were generally conservative: 75.5% used limited or standard templates for dissection. This is in contrast with professional guidelines which recommend extended PLND for greater staging accuracy [25, 26]. This discordance suggests surgeons may weigh increased operative time and risk of complications like lymphocele more than diagnostic yield. We also hypothesize that accumulating evidence demonstrating limited oncologic benefit from extended PLND, including a lack of improvement in biochemical recurrence-free or metastasis-free survival, may influence surgeons to favor a more selective approach when considering the risk-benefit tradeoff. Furthermore, advances in molecular imaging, particularly the increasing use of prostate-specific membrane antigen positron emission tomography/computed tomography (PSMA PET/CT), may contribute to a more individualized approach to PLND.

Variation in perioperative management may be indicative of more risk-averse tendencies and limited resource availability across geographic regions. Routine drain placement (60.6%) and postoperative cystogram (29.9%) were reported at moderately high rates, despite their uncertain utility [27, 28]. Also, nearly 30% of surgeons from outside the US reported that all of their patients undergoing RARP remained hospitalized until day 3 or later. In contrast, US-based surgeons had high rates of discharge by postoperative day 1, as high-volume centers in the US have demonstrated that even same-day discharge can be safe and cost-effective [29, 30]. These differences underscore the impact of healthcare infrastructure and culture in RARP practices.

There are some limitations of this study. First, the relatively small and self-selected cohort may limit the extent to which the observed practice patterns and preferences are representative of the broader international urologic community. Given the method of distribution, this survey may be prone to selection and non-response bias, with responses skewed toward academic surgeons with access to advanced tools and training. Additionally, self-reported data may not reflect actual practice, and the lack of outcome correlation limits conclusions about best practices. Finally, no data were collected regarding the robotic platform used, which limits our ability to determine whether any of the reported approaches or preferences are specific to a particular platform. Still, this study adds to the characterization of global RARP practice patterns. Future research, including qualitative interviews and more granular analyses of geographic and institutional variability, may further elucidate the drivers of RARP practice.

## Conclusion

The current study highlights trends in RARP education, technique, and perioperative management in modern practice. While traditional techniques remain dominant, novel approaches like Retzius-sparing and the Hood technique are less commonly adopted. Training pathways are diverse and often self-directed, which may reflect the need for standardized curricula. Perioperative practices also varied widely. These findings suggest consensus-driven, evidence-based guidelines may help to optimize RARP globally.

## Data Availability

The data that support the findings of this study are available from the corresponding author upon reasonable request, subject to institutional and ethical considerations.
